# Induction of Salivary Proteins Modifies Measures of Both Orosensory and Postingestive Feedback during Exposure to a Tannic Acid Diet

**DOI:** 10.1371/journal.pone.0105232

**Published:** 2014-08-27

**Authors:** Ann-Marie Torregrossa, Larissa Nikonova, Michelle B. Bales, Maria Villalobos Leal, James C. Smith, Robert J. Contreras, Lisa A. Eckel

**Affiliations:** Department of Psychology, Program in Neuroscience, Florida State University, Tallahassee, Florida, United States of America; Barnard College, Columbia University, United States of America

## Abstract

There are hundreds of proteins in saliva. Although it has long been hypothesized that these proteins modulate taste by interacting with taste receptors or taste stimuli, the functional impact of these proteins on feeding remains relatively unexplored. We have developed a new technique for saliva collection that does not interfere with daily behavioral testing and allows us to explore the relationship between feeding behavior and salivary protein expression. First, we monitored the alterations in salivary protein expression while simultaneously monitoring the animals' feeding behavior and meal patterns on a custom control diet or on the same diet mixed with 3% tannic acid. We demonstrated that six protein bands increased in density with dietary tannic acid exposure. Several of these bands were significantly correlated with behaviors thought to represent both orosensory and postingestive signaling. In a follow-up experiment, unconditioned licking to 0.01–3% tannic acid solutions was measured during a brief-access taste test before and after exposure to the tannic acid diet. In this experiment, rats with salivary proteins upregulated found the tannin solution less aversive (i.e., licked more) than those in the control condition. These data suggest a role for salivary proteins in mediating changes in both orosensory and postingestive feedback.

## Introduction

Variation in taste preferences in general, and bitter taste perception in particular, may play a key role in dietary choice, which has an uncontestable effect on human health. Bitter compounds are often thought of as aversive stimuli to be avoided [1] but are also common constituents of the omnivore diet [2]. A large number of nutritionally significant food sources contain bitter phytochemicals (e.g., broccoli [3], spinach [4]), many of which have beneficial value [3], [5]. Understanding the complexity of bitter taste acceptance has been of interest to the research community for decades [6]–[8] and is part and parcel to the development of strategies to influence dietary choices in the service of promoting human health. Studies on bitter taste have focused mainly on the molecular identification of bitter taste receptors and their ligand-binding properties [9], [10] as well as the genetic variation in such receptors [7], [8], [11]. In comparison, very little work has been done to investigate how salivary proteins interact with taste stimuli and alter taste sensitivity despite the fact that, under normal feeding and drinking conditions, taste compounds must inescapably mix with saliva before reaching their receptor targets. This sets the stage for potential modulation of the taste signal at its most fundamental level.

Proteomics suggest that over 1,000 different proteins are present in the saliva of healthy adults [12]. The protein content of saliva is altered under a variety of circumstances including diet composition [13], [14], disease [15], [16], exposure to compounds like capsaicin [17] or bitter taste stimuli [18]–[21]. Likewise, there is evidence that some of these proteins modulate bitter taste acceptance. For example, Kock et al. [22] introduced von Ebner's gland protein, a protein found in rat saliva, to mice that do not normally express the protein. The presence of von Ebner's gland protein altered the acceptability of the bitter stimulus denatonium benzoate. Dsamou et al. [23] determined that another salivary protein, cystatin SN, was related to variation in human sensitivity to the bitter taste of caffeine.

Most studies examining the role of salivary proteins on food intake have focused on the relationship between a class of proteins referred to as proline-rich proteins (PRPs) and a class of plant secondary compounds referred to as tannins. Tannins are polyphenolic compounds commonly consumed by both humans and non-human mammals. In the human diet, tannins are present in foods such as sorgum, tea, red wine, beans and unripe fruits. Tannins are not only associated with a sense of astringency [4], [24], but several polyphenolic compounds classified as tannins activate various human bitter taste receptors, TAS2Rs [25]. In addition to their astringency and bitter taste, tannins are considered “anti-nutritional” because they can reduce non-heme iron absorption, cause loss of endogenous nitrogen, reduce digestibility and damage the kidney, liver, gastrointestinal mucosa and gastrointestinal epithelium (see [21]). Although avoidance of tannins is well documented (see [26]), no studies have addressed whether it is the taste or the postingestive effects of tannins that alter food intake. One study suggested that avoidance of high tannin diets is the result of both conditioned and unconditioned avoidance [27], however, this study could not decouple the oral and gastrointestinal effects of tannin, so the relative roles of these two sources of negative feedback remain unclear.

Salivary PRPs are considered the first line of defense against the negative consequences of ingested tannins [26]. PRPs avidly bind tannins into a complex that is stable even under the conditions of the stomach and intestine [28], [29], and this complex allows the consumer to pass the tannin complex as fecal material. PRPs are not constitutively produced in rodents, as they appear to be in humans (see [30]), but can be induced by exposure to tannin containing diets or via injections of the β-agonist isoproterenol [31]. It has been demonstrated that several species of rodents decrease food intake and lose body mass when fed tannin containing diets in the absence of PRPs. In rats, food intake is reduced for several days before they resume their normal intake, presumably due to increases in PRPs [32]. In support of this hypothesis, it has been demonstrated that hamsters, which do not upregulate PRP production in response to tannin-containing diets, reduce intake on tannin diets indefinitely and lose a life-threatening amount of body mass while on the diet [32].

Although it is generally accepted that PRPs allow animals to consume tannin-containing diets, it is unclear whether the increase in diet acceptability is due to a change in the perception of the tannins in the mouth [30], [33], [34] or due to an ability to protect the gut from negative postingestive effects [35]. Glendinning [33] demonstrated that mice injected with isoproterenol, to increase PRP production, show an increased preference for, and intake of, tannin solutions compared to control mice via increases in both the size and number of drinking bouts (i.e. the amount of time an animal licked on the sipper tube before taking a break). These data imply a modification in the orosensory perception of the tannin solutions.

In order to better understand the role of salivary proteins on tannic acid acceptance, we conducted two experiments. In our first experiment, we monitored the alterations in salivary protein expression, while simultaneously monitoring feeding behavior, in animals consuming a control or tannic acid containing diet. In the second experiment, unconditioned licking of tannic acid solutions was measured during a series of brief-access (30-s) tests conducted before and after exposure to a tannic acid containing diet. This allowed us to isolate the contribution of orosensory signals to ongoing fluid intake and thus provided a direct measure of tannin palatability.

## Methods

### Experiment 1

#### Animals and housing

Animals (male Long-Evans rats, Charles River Breeding Laboratory, Raleigh, NC; weighing 200–240 g at study onset) were assigned to either an experimental or control group. The experimental group (n = 8) was individually housed in custom-designed Plexiglas shoebox cages. Food compartments attached to the front of each cage were equipped with infrared light emitting diodes and photo detectors. Feeding events were detected when the rat's head entered the food compartment and broke the photo beam. The time and duration of each beam break, recorded on a computer, was used to estimate meal size and rate of feeding. Rats were allowed ad lib access to food and tap water. The control group (n = 4) was housed in cages similar to the test cages but without with photo beams. The colony room was maintained at a 20±2°C with a 12∶12 h light/dark cycle. All animal procedures were approved by Florida State University Animal Care and Use Committee (ACUC).

#### Analysis of Feeding Behavior

The start of a meal was defined as a minimum of 3 s of activity in the food cup and individual meals were considered terminated when there was no feeding activity for >5 min. Daily food intake was measured gravimetrically. Average daily meal size was defined as the total (24 h) food intake (g) divided by the number of meals consumed each day. Average daily rate of feeding was defined as the total (24 h) food intake (g) divided by the total amount of feeding activity (min).

#### Saliva collection

Saliva was collected from awake, trained animals. During training, each rat received 2 ml of 30 mM citric acid in 1 M sucrose. This solution, which served as an unconditioned stimulus (US), was pipetted into the mouth daily in 200 µl increments. Animals were held against the experimenter's chest with the palm of the hand while the head and mouth were stabilized by the first two fingers and the thumb. The pipette tip, which served as a conditioned stimulus (CS), was inserted into the side of the mouth and was moved over and under the tongue during CS/US pairings. The animals received 25 pairings of the US and CS across an approximately 20-min period, every day for 12 days. In this way, animals were conditioned to salivate by merely inserting the pipette into the mouth. Following training, we began collecting salivary secretions on alternate days. Saliva was gently aspirated from below and around the tongue using a 200 µl pipette fitted with a wide orifice tip. We stimulated the animals with the US several times during the saliva collection. After stimulation we allowed the rat to rest for ∼60 s to minimize contamination of the saliva with the solution. We used this procedure to collect all of the saliva in Experiment 1. We now know that this intermittent stimulation is unnecessary and in Experiment 2 we were able to collect saliva from animals without the presence of the US on collection days. Both of these methods allowed us to collect uncontaminated saliva from sublingual, submandibular and parotid secretions, as all are stimulated by sweet and sour mixtures [36].

Saliva samples, ranging from 50–100 µl were immediately placed on ice in 10 µl of 10x Halt protease/phosphatase inhibitor cocktail (Thermo Scientific). All samples were then frozen at −20°C for later analysis. Saliva was collected from half of the rats on odd test days while the other half received only the citric acid/sucrose mixture and the reverse was conducted on even days. Thus, saliva was collected representing each day of the trial.

#### Diets

All rats were maintained on Purina 5001 and tap water ad lib until study onset at which point all rats were given a purified control diet ad lib (diet modeled after [28], Table 1). The control group was maintained on the control diet during the entire course of the experiment. The experimental group was fed the control diet for 12 days followed by the tannic acid diet for 15 days. The tannic acid diet contained 3% tannic acid (Sigma Aldrich, lot number SZBC0460V) by weight and 3% less cellulose but was otherwise identical to the control diet. The first saliva samples were collected after the animals were maintained on the control diet for 4 days.

**Table 1:** Composition of the control and 3% tannic acid diets used in Experiments 1 and 2. These diets were based on Skopec et al. [28].

Ingredients	Control Diet (g/kg)	3% Tannic acid diet (g/kg)
Casein	200	200
DL-Methionine	3	3
Sucrose	500	500
Corn starch	150	150
Corn oil	50	50
Cellulose	50	20
Tannic acid	0	30
Mineral Mix, AIN-76 Teklad	35	35
Vitamin Mix, AIN-76	10	10
Choline bitartrate	2	2

#### High Performance Liquid Chromatography (HPLC) analysis of tannic acid

HPLC analysis was performed in the Florida State University Department of Biology Analytical Laboratory. A sample of the tannic acid that was used in the diet (Sigma Aldrich, lot number SZBC0460V) was dissolved in 20% acetonitrile/0.1% TFA at a concentration of 5 mg/ml. Two µl of this solution was loaded onto a C18 reversed phase column (Agilent Zorbaz 300SB-C18, 4.6×250 mm, 5 micron) equilibrated in 20% acetonitrile/0.1% TFA. Tannins were eluted with a 10 min linear gradient to 40% acetonitrile/0.1% TFA, at a flow rate of 1 ml/min and detection at 280 nm. Pentagalloyl glucose (PGG, Sigma Aldrich, lot number 063M4713V PCode 71813) was dissolved in 20% acetonitrile/0.1% TFA at a concentration of 5 mg/ml, and 0.25 µl of this solution was analyzed by reversed phase. In addition, a tannic acid sample spiked with PGG standard was analyzed to confirm the identification of PGG in the tannic acid mixture. All HPLC analyses were preformed on a Beckman System Gold HPLC equipped with a 168 diode array detector, 126-solvent delivery system, and 32 Karat software for data analysis.

#### Salivary protein processing

Saliva samples were defrosted and mixed with equal volumes of ice-cold 20 mM Hepes buffer, pH 7.4, supplemented with 2x Halt protease/phosphatase inhibitor cocktail (Thermo Scientific) and 2 mM phenylmethylsulfonyl fluoride on the day of processing. Samples were centrifuged at 2000xg for 6 min at 4°C to remove cells and debris. The supernatant was aspirated and was thereafter referred to as whole saliva. Total protein concentration was determined by the bicinchoninic acid (BCA) protein assay method (Pierce Protein Biology Products).

#### Gel electrophoresis and Western blotting

Equal volumes of saliva sample were mixed with 1/4^th^ volume of 4x Invitrogen sample buffer with reducing agent, heated at 70°C for 10 min and resolved on a 12% SDS-PAGE (Invitrogen) with MOPS buffer. Molecular mass markers (Invitrogen, Ref LC5800, or BioRad, Ref 161-0374) were run simultaneously with the samples in each gel to calibrate molecular masses of each protein band. Gels were fixed in 40% methanol in 10% acetic acid for 30 min followed by staining with Coomassie Brilliant Blue R250 (0.1% stain in 25% methanol and 10% acetic acid) for 1 h. These gels were distained in 10% acetic acid according to previously published protocols [37], [38]. Although it is usual to rinse gels with methanol/ethanol in the destaining solution, omitting these chemicals distinguishes PRPs, which stain pink-violet, from other proteins, which stain blue [37], [38]. These data were used to determine which bands contained PRPs. Bands were captured using Kodak Imaging Station 440 CF (Kodak, Rochester, New York). Densitometric analysis was performed using Kodak 10 Imaging Analysis software (Kodak).

Secretory immunoglobulin A (IgA) was used as a protein loading control. Preliminary experiments demonstrated that IgA expression, in samples loaded at the same protein level, was unchanged by our treatment. Salivary IgA levels were estimated by immunoblotting using goat polyclonal antibody to rat immunoglobulin A. We used 1/10 volume of each saliva sample used for Commasie staining but without the reducing agent. Following electrophoresis, proteins were transferred to Hybond-P Western blotting membranes (VWR Scientific Product). The blots were blocked for 1-h at room temperature with 5% BSA in Tris-buffered saline containing 0.1% Tween 20 (TBST). The membranes were then incubated for 1-h at room temperature with goat anti-IgA antibody (1: 80 000) conjugated with horseradish peroxidase, (Abcam Inc. # ab97185). The immunoblotted proteins were detected using Super Signal West Dura Extended Duration Substrate (Thermo Scientific, #34076) and CL-XPosure Film (Pierce Protein Biology Products). Following development of chemiluminescence film, images of bands were captured using a Kodak Imaging Station 440 CF (Kodak, Rochester, New York). Densitometric analysis was performed using Kodak 10 Imaging Analysis software (Kodak) that measures the optical density of the immunosignal.

To quantify protein level, each gel contained several control saliva samples from untreated animals. Band intensity was normalized using the corresponding IgA protein signal and expressed as relative amount to average intensity of the control sample, which was assigned a value of 1.

#### Detection of glycosylated proteins

Because glycosylated PRPs are less effective at binding tannins than basic PRPs [39], we used the Pro-Q Emerald 300 Glycoprotein Gel and Blot stain Kit (Molecular Probes # P21857) to determine if the proteins were glycosylated. The kit detects as little as 0.5 ng of glycoprotein per band. Saliva samples from animals in our experimental group were tested to demonstrate the likelihood of glycosylation of each protein band. Samples (6 µg) were loaded in duplicate on a single 12% SDS/PAGE gel. After resolving the gel, it was cut in two. Each half contained several concentrations of protein collected on several days of the study from a single animal and Candy-Cane glycoprotein molecular weight standards, which include glycosylated and non-glycosylated proteins. One half was stained using Pro-Q Emerald 300 glycoprotein detection kit, which detects only glycosylated proteins. The other half was stained with Coomasie stain to detect total protein.

#### Protein identification

Amino-acid sequencing was performed at the Florida State University Translation Science Laboratory. Following electrophoresis, the gels were fixed and Coomassie-stained. Protein bands of interest were excised followed by distaining, reduction, alkylation, dehydration and in-gel tryptic digestion. Tryptic digestion was conducted using the Calbiochem ProteoExtract All-in-One Trypsin Digestion Kit (Merck, Darmstadt, Germany) according to the manufacturer's instructions and using LC-MS grade solvents (Avantor Performance Materials, Center Valley, PA, USA). The digestion supernatant was stored at −80°C prior to analysis. Nanospray LC/MS^E^ was done using a Synapt G2 HD Mass Spectrometer equipped with an integrated *nano*Acquity UPLC for on-line chromatographic separation of tryptic peptides prior to MS analysis (Waters Corp., Milford, MA, USA). Samples were adjusted to 3% acetonitrile (ACN) in LC-MS grade water with 0.1% formic acid (FA) and 25 fmol/µl rabbit Phosphorylase B (PhosB, Waters Corp.) as an internal standard. Glufibrinopeptide (785.8426 m/z, Waters Corp.) was used as the lock mass (external calibrant). Instrument data acquisition parameters are provided in Table S1. Raw data were generated using MassLynx version 4.1 software (Waters Corp.) and data were processed in ProteinLynx Global SERVER version 3.0. After analysis of the protein sequences of the 14 kDa band, it was identified using the PLGS Identity search algorithm, which queried a Rattus norvegicus protein databank containing sequences from the Uniprot Protein Knowledgebase (www.uniprot.org) and the rabbit PhosB sequence. Results were validated by manual review of the raw data and results.

#### Statistical Analysis

Feeding data were analyzed using repeated-measures ANOVAs. To evaluate group differences in protein expression, protein levels from experimental and control animals were compared using ANOVA with diet as a between-subjects factor and time as a within-subjects factor. Significant ANOVA effects were further evaluated using Bonferoni corrected paired t-tests. Because saliva was collected from half of the rats each day, odd and even days were combined for each sampling period so that all animals were represented in the same repeated measures analysis. A linear mixed model analysis was used to explore the associations between feeding behavior and the protein measures while accounting for the correlations associated with the repeated-measures factor. We limited this analysis to the feeding behavior recorded and salivary samples collected during the first four days of tannic acid diet exposure. This time frame represents the dynamic phase when salivary proteins were upregulated and changes in feeding behavior were most pronounced. ANOVAs and post-hoc tests were conducted in Systat 12. Linear mixed models were run in SPSS 19.

### Experiment 2

#### Animals and housing

Male Long Evans rats (n = 12) were individually housed in custom designed Plexiglas shoebox cages, equipped with a feeding niche that provided access to a spill-resistant food cup. The colony room conditions were identical to that described above. Rats were allowed ad lib access to diets (control or 3% tannic acid, lot number SZBA 3060V, Sigma) and tap water, except where otherwise noted.

#### Analysis of Licking Behavior

To determine whether induction of PRPs affects the rats' taste responses to tannic acid, we recorded the unconditioned licking responses to varying concentrations of tannic acid in a test chamber designed to measure solution licking (Davis MS80 Rig; Dilog Instruments and Systems, Tallahassee, FL). This apparatus consisted of a Plexiglas cage with a wire mesh floor. An opening at the front of the cage allowed access to one of ten spill-proof glass drinking tubes that reside on a sliding platform. A mechanical shutter opened and closed to allow the rat access to one of the ten tubes for a user-specified length of time. A computer controlled the movement of the platform, order of tube presentation, opening and closing of the shutter, duration of tube access and interval between tube presentations. Each individual lick was detected by a contact lickometer and recorded on a computer via DavisPro collection software (Dilog Instruments and Systems).

Rats were adapted to the test chamber and trained to drink from the sipper tubes for 4 days. On the first day of training, rats were 20-h water-deprived. The rat was presented with a single stationary bottle of 0.25 M sucrose for 30 min. On the second day, a single tube containing 0.25 M sucrose was presented again, however, the rat had to lick the tube 50 times in order for the training program to begin. At the start of the program, the shutter closed for 10 s before a new tube, containing 0.25 M sucrose, was presented. The rat was given 180 s to initiate licking and once licking was recorded the rat was given 30-s access to the tube. At the conclusion of either the 30-s access or the 180-s limit, the shutter was closed again for 10 s. Each of the 10 tubes, all containing 0.25 M sucrose, was presented 3 times. The entire training program took an average of 15 min.

During testing rats were 20-h water deprived. Rats were given varying concentrations of tannic acid (0.0, 0.011, 0.02, 0.046, 0.09, 0.187, 0.375, 0.75, 1.5, and 3%). Each bottle was presented three times per session and sessions were organized in randomized blocks. Identical to training; the rat was given 180 s to initiate licking, 30-s access once a lick was recorded and a 10-s delay between stimulus presentations.

All rats were tested in the brief-access taste test while maintained on the control diet. After initial testing, rats were assigned to either a control group (n = 6), which received only the control diet prior to the second brief-access taste test, or an experimental group (n = 6), which was maintained on the 3% tannic acid diet prior to the second brief-access taste test.

#### Saliva collection

As described above, animals were conditioned to salivate when a pipette was inserted into their mouth. During training, animals received 5 ml of 30 mM citric acid in 1 M sucrose pipetted into the mouth daily for 14 days in 200 µl increments. Following training, conditioning was maintained by pipetting the solution into the animals' mouths once a week until the conclusion of the experiment. We found that we had no difficulty maintaining the conditioning across these time scales. Because this testing paradigm required the animals to be water deprived when saliva was collected, all saliva collection was conducted in the absence of the citric acid mixture; the conditioning alone was sufficient for the procedure. Saliva was collected the day before the brief-access test while animals were water replete and the day of the brief-access test while animals were 20-h water deprived.

#### Salivary protein processing

Salivary samples were processed as described above with one exception. In Experiment 1, we used IgA to confirm our total protein measures, but it was not possible to do this in the water-deprived condition. Previous studies have shown that expression of IgA is altered by water deprivation [40] as is protein concentration of saliva [41]. Because we cannot be sure that deprivation alters the expression of all proteins equally, we used a more conservative approach of analyzing the salivary protein data by t-test comparing differences in protein expression between the two test sessions (i.e. test 1 protein expression - test 2 protein expression) by treatment (control fed vs. tannin fed). Because these data do not allow an impression of relative abundance, we also present the data as corrected to the water replete expression of the same protein. We do not compare these data statistically but provide them merely to illustrate the relative abundance of the proteins.

#### Statistical Analysis

The behavioral data presented represent the mean number of licks to each concentration and the curves fitted to the mean number of licks to each concentration. Curves were fit to the lick data (excluding the water licking) of each individual animal using a 3-parameter logistic function: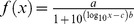 in which a =  asymptotic lick response, excluding water; b =  slope; and c =  log_10_ concentration at one-half of the asymptote (i.e., EC_50_). Licking behavior was compared by repeated-measures ANOVA and Bonferoni corrected paired t-tests. The curve parameters were compared using Bonferoni corrected paired t-tests. Curves were fit to the data and ANOVAs were conducted in Systat 12.

## Results

### Experiment 1

#### HPLC analysis of tannic acid

The area under the curve was calculated based on the chromatograms from the HPLC analysis. These data revealed that the ∼67% of the area under the curve was identical to, or to the right of, the peak caused by our additional PGG marker. Larger tannin compounds are known to better interact with PRPs compared to smaller compounds [42], because PGG is a large tannin compound known to interact with PRPs, we have used PGG as a marker for size in this study. These data suggest that 67% of compounds in the tannic acid, are the same size or larger than PGG (Figure S1), therefore, we believe that the tannic acid supplied by Sigma contains large tannin compounds, which are capable of interacting with salivary proteins.

#### Induction of salivary proteins

**Figure 1:**
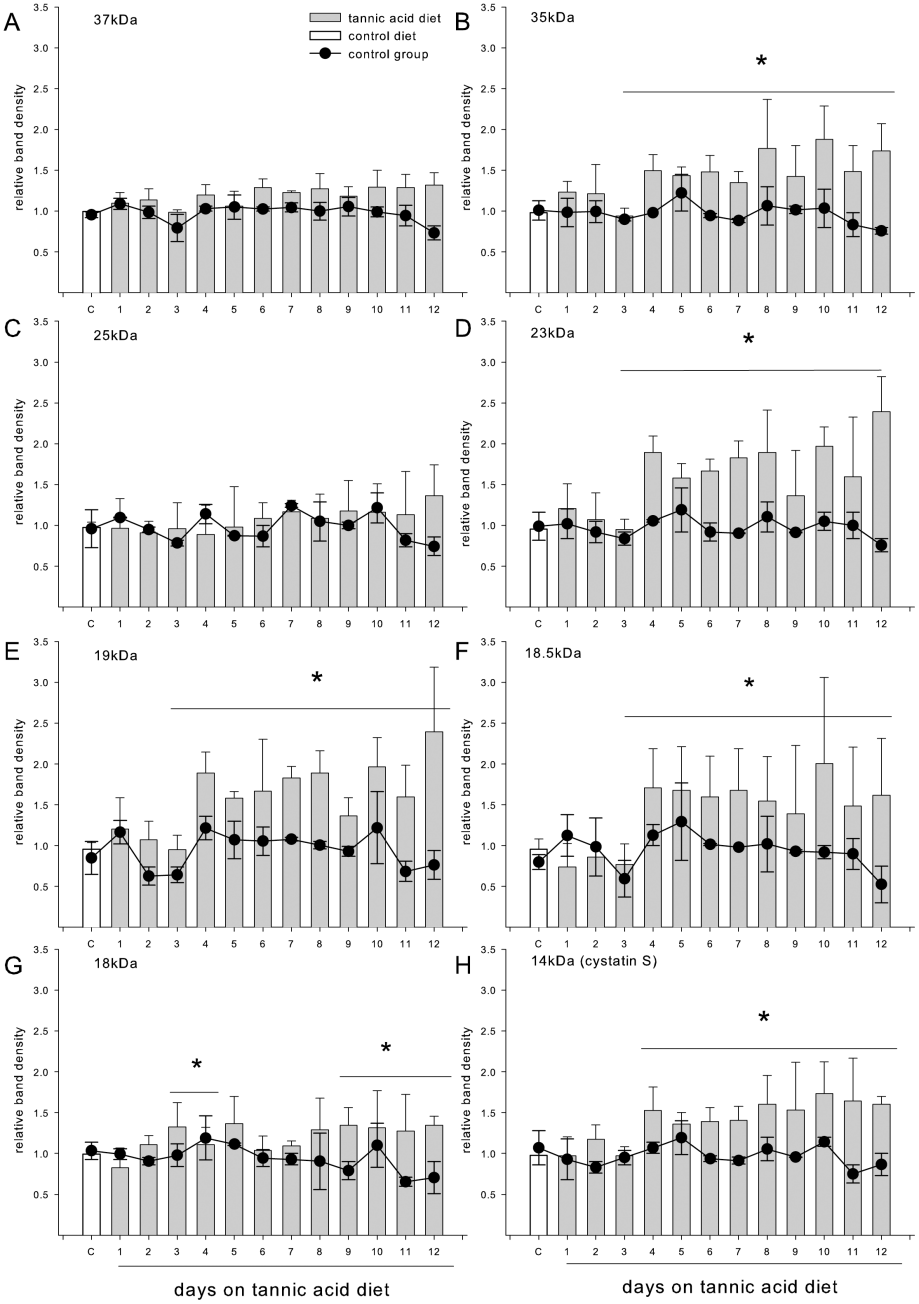
Data are densitometry units normalized to average control diet protein expressions (set to 1) as well as IgA expression. White bars represent protein expression measured while the experimental animals were consuming the control diet (average of the final 5 days; depicted by white bars labelled “C”), and grey bars represent expression of the same protein while the experimental animals were consuming the 3% tannic acid diet. The line graphs represent the same protein densitometry measures for animals that were maintained on the control diet for the entire course of the study. *Significant within-subject difference between protein expression on the control and experimental diets (p<0.05, bonferoni corrected for multiple comparisons).

**Figure 2:**
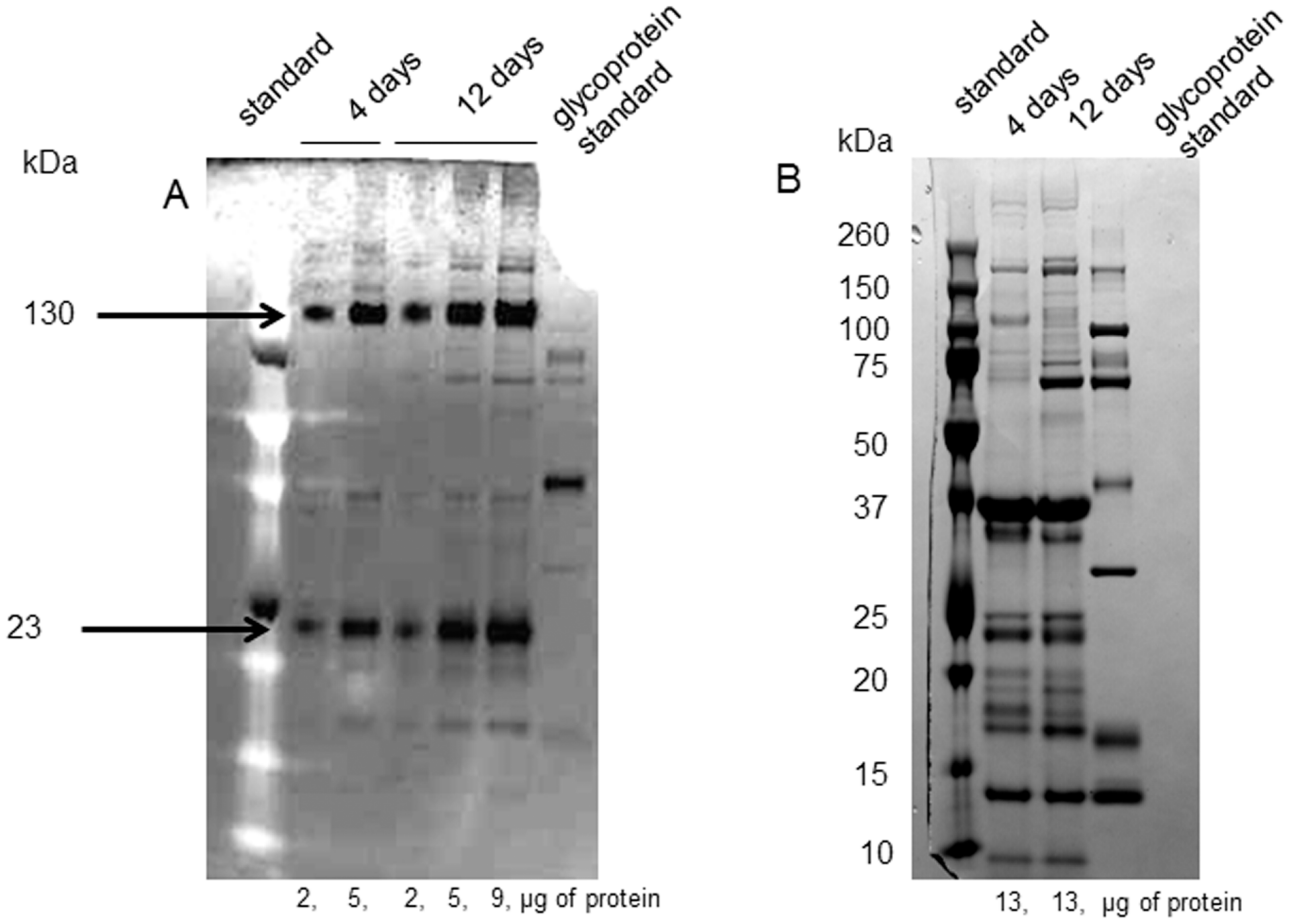
Representative samples of the glycosylation determination. The gel contains saliva samples from a single rat after 4 or 12 days exposure to the tannic acid diet. The samples were loaded in duplicate on the same12% SDS/PAGE. The quantity of protein loaded in each lane was varied to allow for maximal visibility of the protein band. After resolving the gel, it was cut into two pieces. Gel A was stained using Pro-Q Emerald 300 glycoprotein detection kit, which detects only glycosylated proteins. Gel B was stained with Coomassie R250 stain, which detects total proteins. Both gels contain both Candy-Cane glycoprotein molecular weight standards and Bio-Rad molecular weight standards.

In the experimental group, we saw a trend toward increased protein concentration with exposure to the tannic acid diet, but it failed to reach significance (F_9,63_ = 2.0, p = 0.06). In the control group there was no difference in the total protein concentration across time (F_5,20_ = 1.9, p = 0.13). Our method separated out 12–15 bands from each sample of saliva (Figure S2). We quantified the protein level of all the major bands identified as PRPs (37 kDa, 35 kDa, 23 kDa, 18.5 kDa and 19 kDa) and three non-PRP bands (25 kDa, 18 kDa and 14 kDa) that appeared to be altered by the tannin diet. Quantification confirmed that the density of 6 of the 8 bands was increased by tannic acid exposure. ANOVAs revealed a significant interaction between group (tannin or control treated) and time for the PRPs at 35 kDa, 23 kDa, 18.5 kDa,19 kDa, and the non-PRPs at 18 kDa and 14 kDa, Table 2, Figure 1). Two proteins on the gel were identified as glycosylated (23 and 130 kDa), only one of these, the 23 kDa band, was significantly modified by the diet treatment (Figure 2).

**Table 2:** Summary of ANOVAs comparing normalized densitometry units of protein bands at each of the listed masses (kDa).

	between subjects			within subjects			interaction effects		
kDa	df	F	p	df	F	p	df	F	p
37	1,10	3.047	0.111	6,60	1.675	0.143	6,60	2.1	0.065
35	1,9	10.86	0.009*	6,54	2.9	0.016*	6,54	3.17	0.01*
25	1,10	0.54	0.49	6,60	1.79	0.116	6,60	1.92	0.084
23	1,9	16.27	0.003*	6,54	4.1	0.002*	6,54	4.6	0.001*
19	1,10	12.35	0.006*	6,60	3.26	0.0088*	6,60	3.4	0.006*
18.5	1,10	3.7	0.082	6,60	1.9	0.091	6,60	2.4	0.034*
18	1,10	12.5	0.005*	6,60	2.264	0.383	6,60	2.26	0.049*
14	1,10	17.13	0.002*	6,60	3.5	0.005*	6,60	4.2	0.001*

Analyses were conducted with experimental group (control or tannic acid diet) as the between-subjects comparison and time (days) as the within-subjects comparison. *ps<0.05.

Several bands seemed to be composed, to varying degrees of both PRP and non-PRP proteins as the upper portion of the band was stained pink, while the lower portion of the band was stained blue or vice versa (e.g., 23 and 18 kDa). In these cases, we could not segregate the colors during density counts. It is unsurprising to find several proteins in a single band, for example Mirels et al. [43] previously described an 18.5 kDa band isolated from rat submandibular and sublingual saliva consisting of several proteins. We were unable to determine protein sequences from the bands that were identified as proline-rich or appeared to contain some PRPs, due to the heterogeneity of sample.

**Figure 3:**
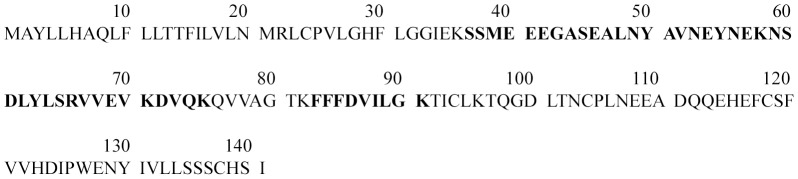
The sequence of rat protein cystatin S (Uniprot accession P19313). Bold regions indicate where tryptic peptides detected in proteomic analysis overlay the protein sequence.

We were however, able to identify the dominant protein in the 14 kDa band. This band steadily increased across days of dietary exposure and was maximally expressed by the 4^th^ day of dietary exposure. Proteomic analysis of the 14 kDa band identified rat cystatin S (Uniprot accession P19313) as the dominant protein present. Cystatin S was identified with >95% confidence and high confidence tryptic peptides combined together provided 34% coverage of the cystatin S sequence (Figure 3), indicating that the dominant protein in the band was a cysteine protease inhibitor present in the saliva of rats and humans [44]–[46].

#### Behavioral data

For all the feeding behavior measures, we used the average of the final five days on the control diet as our baseline because there were no differences in 24-h food intake (F = _4,28_ = 1.2, p = 0.34), meal size (F = _4,28_ = 0.89, p = 0.48), meal number (F = _4,28_ = 1.38, p = 0.27), or rate of feeding (F = _4,28_ = 0.23, p = 0.92).

#### Total intake

**Figure 4:**
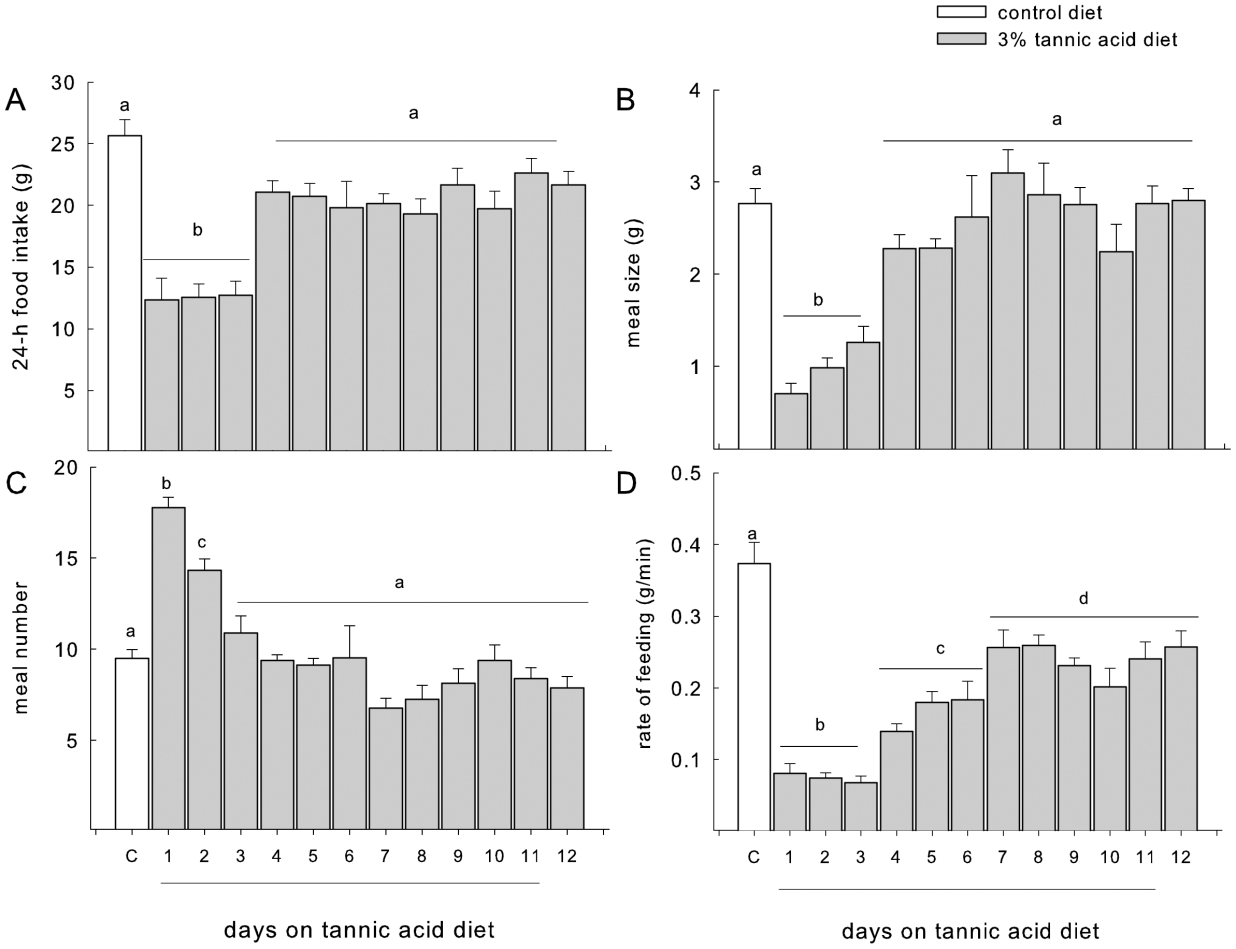
A–D: White bars represent feeding behaviors measured while animals were consuming the control diet; grey bars represent the same behaviors measured while animals were on the 3% tannic acid diet. The white bar labeled ‘C’ represents a 5-day average of behavioral measures on the control diet prior to exposure to the tannic acid diet. Food intake (A) and meal size (B) were decreased on the first 3 days of exposure to the tannic acid diet but returned to control levels by day 4. Meal number (C) was increased during the first 2 days of exposure to the tannic acid diet but returned to control-levels by day 3. Rate of feeding (D) was decreased throughout the entire exposure to the tannic acid diet buy this effect was most pronounced during the first 3 days.

In comparison to baseline, rats reduced total intake when fed a diet containing tannic acid (F = _10,70_ = 12.52, p<0.001, Figure 4a). The largest reduction (∼50% reduction) was seen during the first 3 days of the diet. Total food intake recovered to baseline by the fourth day.

#### Meal size

The reduction in total intake of the tannic acid diet was due primarily to a reduction in meal size (F = _10,70_ = 13.23, p<0.001, Figure 4b). The reduction in meal size was limited to the first three days of tannic acid diet exposure, averaging 75% less than control on the first day. During the following days the meal size returned to baseline levels. The alterations in meal size were significantly related to the expression of the 35, 19, 18.5 kDa bands and cystatin S (see Table 3), but not to the expression the 23 or 18 kDa bands (Table 3).

**Table 3:** Summary of linear mixed model analyses.

	meal size			number of meals			rate of feeding		
kDa	df	F	P	df	F	P	df	F	P
35	1,16	5.07	0.04*	1,16	1.01	0.33	1,15.8	3.03	0.1
23	1,16	3.33	0.09	1,16	0.06	0.8	1,15.6	4.6	0.05*
19	1,15.6	5.5	0.03*	1,16	2.9	0.11	1,16	1.85	0.2
18.5	1,16	3.04	0.01*	1,16	3.21	0.09	1,11.1	5.5	0.04*
18	1,16	1.26	0.278	1,16	0.318	0.58	1,16	0.2	0.65
14	1,16	7.4	0.02*	1,16	2.6	0.12	1,12.2	2.8	0.12

We looked for the predictive value of each of the normalized densitometry units of protein bands at each of the listed masses (kDa) on feeding behaviors. Analyses were restricted to the first four days of exposure to the tannin diet as this represented the dynamic phase over which changes in feeding behavior and salivary protein expression were observed. *ps<0.05.

#### Meal number

Rats increased the number of meals consumed during the first 2 days of the tannic acid diet by 70% (F = _10,70_ = 13.3, p<0.001, Figure 4c) but returned quickly to baseline levels. The increase in meal number was likely acting to increase total intake in the face of the sharp decrease in meal size. These changes were uncorrelated with the increase in any of the proteins measured (Table 3).

#### Rate of feeding

Rats displayed a decrease in the rate of feeding while on the tannic acid diet (F = _10,70_ = 35.84, p<0.001, Figure 4d). This decrease was greatest during the first 3 days and then stabilized by day 7 at a level that was significantly lower than baseline. Alterations in rate of feeding were significantly related to the expression of the 23 and 18.5 kDa bands (Table 3), but not to the expression of the other measured protein bands (Table 3).

#### Body Mass

Rats lost a significant amount of body mass after the first night on the tannic acid diet (356.9±8.9 to 345.8±7.1 g, t_7_ = 4.9, p = 0.002), Animals did not continue to lose significant amounts of body mass, but did decrease the rate in which they gained weight while being fed the tannic acid diet. Weight gain during the last 3 days of the control diet was 12.9 g (±1.8), however, weight gain dropped to 1.3 g (±2.0, t_7_ = 4.2, p = 0.004) on the first 3 days of the tannic acid diet. The effect on body mass was, however, transient and animals had regained sufficient weight by the fourth day of the tannin treatment that they no longer differed from the last day of the control diet (t_7_ = 1.6, p = 0.14).

### Experiment 2

#### Induction of salivary proteins

**Figure 5:**
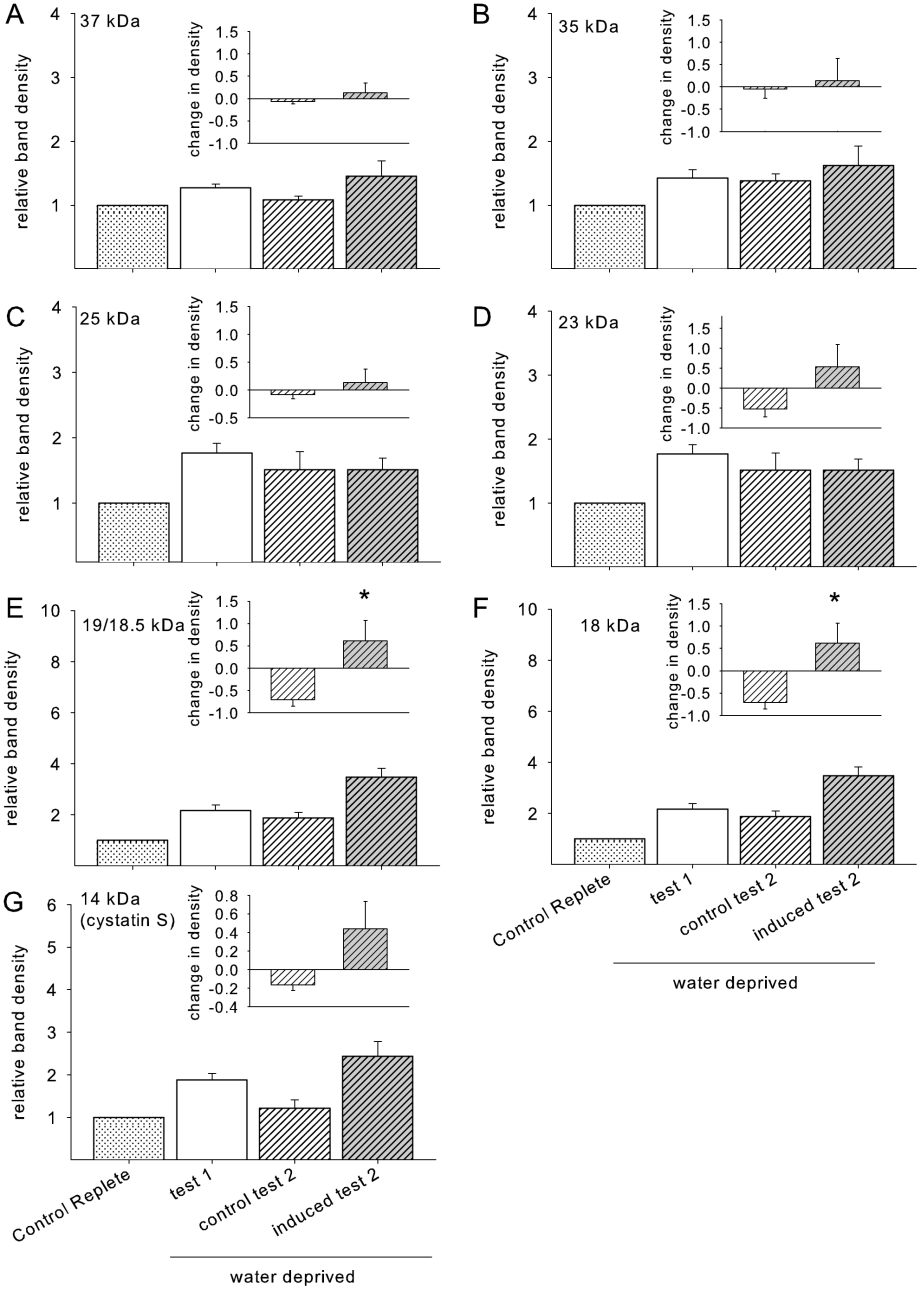
Data depicted in the larger graphs are densitometry units normalized to average control (water replete) protein expressions (which are set to 1). The first bar (stippled) represents the average water replete expression of protein concentration, while all rats were fed the control diet. The data in the remainder of the bars were collected in the water-deprived condition. The white bar represents the average protein expression of all rats on the day of their first exposure to the brief-access taste test. The white bar with hash marks represents the saliva samples collected the day of the second exposure to the brief-access test by rats that were maintained on the control diet. The gray bar with hash marks represents the saliva samples collected the day of the second exposure to the brief-access test by rats that were maintained on the tannic acid diet between exposures. Statistical analyses were not preformed on these data. Total protein concentration was significantly altered by water deprivation making comparisons between samples unreliable. We have presented them only to illustrate the relative abundance of proteins across treatments. The inset graphs represent the change in densitometry units between the two test sessions (i.e. test 1 protein expression- test 2 protein expression) for the control group (white bar with hash marks) and experimental group (gray bar with hash marks). *Experimental group greater than the control group, P<0.05.

As expected, salivary protein concentration was significantly increased by water deprivation (F_3,18_ = 6.09, p = 0.005) making comparisons between water replete and deprived samples unreliable. In this experiment, band densities were such that we were unable to distinguish between the 19 and 18.5 kDa bands, therefore we calculated these proteins as a single band. Since most of the proteins of interest seem to be upregulated by water deprivation alone (Figure 5), we therefore compared only the water deprived samples as described in the methods. The 19/18.5 kDa band density was significantly increased by the diet treatment compared to the control group (Figure 5, t_8.0_ = −3.28 p = 0.01) as was the non-PRP 18 kDa band (t_8.0_ = −3.32 p = 0.01). There was a non-significant trend for cystatin S to increase (t_8.0_ = −2.13 p = 0.065) and no other proteins were upregulated by the diet treatment (all p-values >0.3).

#### Analysis of licking behavior

**Figure 6:**
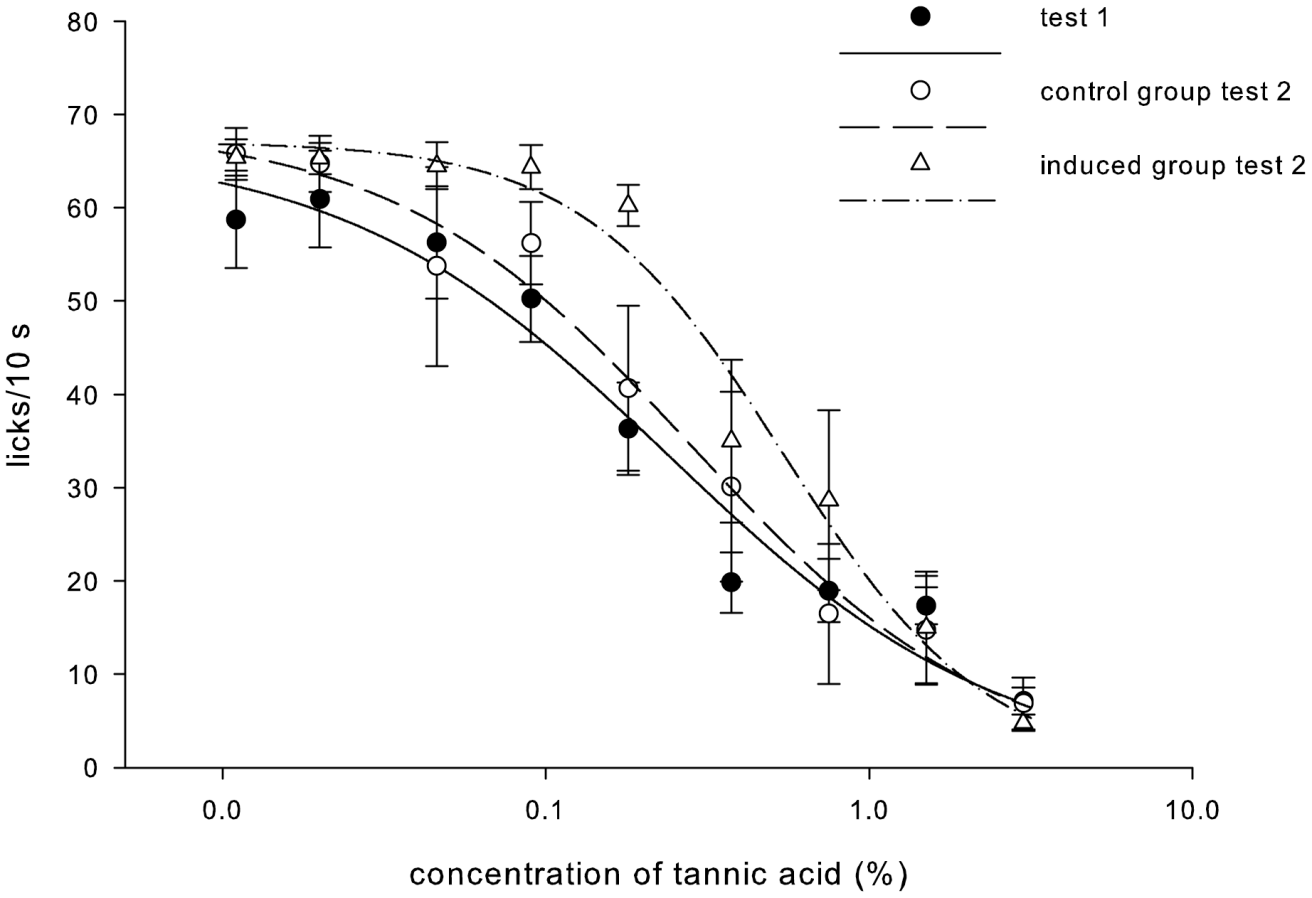
The closed circles represent the average licking of the two test groups during their first exposure to the brief-access test (unconditioned licking does not differ between the groups at this time point p>0.05). The open circles represent the average licking during the second exposure to the brief-access test by rats that were maintained on the control diet. The open triangles represent the average licking during the second exposure to the brief-access test by rats that were maintained on the tannic acid diet between exposures. Lines represent curves fit to the average licking behavior. Rats with an increase in the salivary protein at 19/18.5 and 18 kDa bands show a right-ward shift in the licking response curve demonstrating that they found the tannic acid less aversive in the second exposure than the first exposure, while rats maintained on the control diet did not alter their licking behavior on the second exposure.

The two groups of animals did not differ in their initial licking behavior under control conditions (F_1,10_ = 0.5, p = 0.49). The rats that continued on the control diet did not alter the number of licks upon second exposure to the test paradigm (Figure 6, F_1,10_ = 0.2, p = 0.67). In contrast, rats exposed to the tannic acid diet displayed a strong tendency to increase the number of licks/10 sec (F_1,10_ = 4.5, p = 0.055). To address how the dietary treatment and subsequent salivary changes may have altered the animals' experience with the tannic acid solutions, we compared the EC_50_ of the groups between the first and second Davis Rig exposure. The EC_50_ represents lateral shifts in the curve. While the EC_50_ in the control group did not change between the first and second exposure (t_5_ = 1.4, p = 0.23), there was a rightward shift of the curve in the experimental group (t_5_ = −2.8, p = 0.04) indicating that the tannin was perceived as less aversive in the second licking test conducted following upregulation of salivary proteins.

## Discussion

We demonstrated that the expression of six salivary proteins increased in rats exposed to a tannic acid diet and that the expression of five of these proteins, four PRPs and a non-PRP identified as cystatin S, was significantly correlated with changes in the pattern of food consumption. We also demonstrated that rats increased unconditioned licking to (and thus acceptance of) tannic acid solutions after an upsurge in salivary proteins. Taken together, our findings suggest an important role for these salivary proteins in the control of feeding behavior that is related to their ability to modulate the postingestive and orosensory feedback associated with the consumption of a tannin-rich diet.

### Orosensory Function

Glendinning [33] demonstrated that injection of isoproterenol, and the resulting upregulation of salivary PRPs, increased the acceptability of tannic acid solutions in mice. Glendinning suggested that the PRPs modified the orosensory signal elicited by the tannic acid since mice with upregulated salivary PRPs licked the tannic acid solutions for a longer duration before pausing (i.e., they displayed longer drinking bouts) [33]. Our methods differed from those used by Glendinning as we measured the induction of salivary proteins while the animals consumed the tannin-rich diet in a drug-free state. This allowed us to elucidate the time-course of diet-driven production of salivary proteins. To our knowledge, our study is the first to demonstrate a relationship between salivary PRPs and tannic acid consumption in a normal feeding situation in rodents. Our work provides support for the hypothesis that salivary proteins influence the animals' orosensory response to tannic acid. This was first reflected by a change in the rate of feeding (Experiment 1), a measure indicative of palatability [47], [48]. Protein expression of PRPs of 23 and 18.5 kDa were significantly correlated with an increased rate of feeding while on the tannic acid diet; these data suggest that PRPs played a role in enhancing the diet's palatability. In the brief-access taste test (Experiment 2), the stimulus concentration-lick rate response function to tannic acid solutions was shifted to the right in rats fed the tannic acid diet (Figure 6). This demonstrates that rats found tannic acid less aversive during the second exposure period compared to the first. Conversely, rats fed the control diet throughout the experiment did not alter their unconditioned licking responses during the second licking test. Thus, exposure to the tannic acid diet, not a repetition of the brief-access taste test, was responsible for the shift in licking behavior. Saliva was also collected from water-deprived rats fed the tannic acid and control diets and compared between the two dietary exposure periods for the brief-access taste tests. This analysis revealed that salivary levels of the 19 kDa and the 18/18.5 kDa mixed-protein band increased between the first and second dietary exposure period only in rats fed the tannic acid diet. Because any postingestive effects of the tannic acid solutions were minimized by the brief (30-s) access licking test, this finding suggests a role for these PRPs in the alteration of tannic acid acceptance driven primarily through orosensory control.

While it seems clear from both experiments that the salivary protein band measured at 18.5 kDa (or as 19/18.5 in Experiment 2) is correlated with orosensory acceptance, it is surprising that the 23 kDa band that was significantly predictive of orosenory acceptance in Experiment 1 did not appear to be upregulated in Experiment 2. It should be noted, however, that saliva samples were collected from water deprived animals in the second experiment and not the first, and the lot of tannic acid differed between the two experiments, perhaps these differences may offer potential explanations. Salivary levels of several proteins may be elevated by water deprivation alone (which is necessary to motivate the animals to lick potentially aversive stimuli during brief-access tests) and thus cannot be elevated further by dietary treatment. Unfortunately, due to the wide spread changes in salivary protein expression after water deprivation [41], levels of protein were not directly comparable between water deprived and replete conditions, so this supposition remains speculative. Furthermore, tannic acid is a variable stimulus, one cannot rule out differences in the composition of the stimulus may have altered the patterns of protein upregulation.

Taken together, the data from Experiment 1 on feeding behavior and Experiment 2 on brief-access licking provide evidence that PRPs were related to measures of orosensory signaling. It has been shown that PRPs bind directly to tannins (see [49]). One possibility is that once bound to a PRP, tannic acid is less able to interact with bitter taste receptor proteins and initiate the signaling cascades associated with bitter taste signaling. In this case, PRPs would exert the greatest effect and compromise orosensory signaling the most when tannic acid concentrations are relatively weak in the intermediate concentration range. Indeed, this was the case in Experiment 2 where the largest shift in licking behavior occurred at intermediate tannic acid concentrations. In this experiment, rats responded to the weakest tannic acid concentrations as similar to water and consequently salivary proteins were without effect. At the upper end of the stimulus concentration range, the tannic acid concentration may have been sufficiently strong and salivary protein levels may have been insufficient to prevent access to orosensory receptors. However, at intermediate tannic acid concentrations, PRP levels were sufficient to counteract the bitter taste of tannic acid and alter licking responses.

### Postingestive feedback

Meal size is considered a measure of postingestive feedback, and it has been suggested that animals consuming a bitter compound should halt food intake before surpassing a critical threshold of the possibly toxic compound [50]. Our feeding study demonstrated that, in the presence of tannic acid, rats decreased food intake via a selective decrease in meal size. Over time, rats recovered to baseline intake by a recovery in meal size. This increase in meal size was significantly correlated with the increase in expression of several PRPs (35, 19 and 18.5 kDa) and cystatin S. Although the role of PRPs is unknown in the gut, there is evidence in the literature to suggest that PRPs may be active in the gut. Cai and colleagues [51], [52] have shown that PRPs decrease the absorption of tannins across intestinal cells demonstrating that PRPs modulate interactions between tannic acid and the intestinal environment.

Our current findings suggest that additional research is needed to investigate the potential roles of PRPs and cystatin S in modulating postingestive signals arising from the consumption of bitter compounds. In this regard, previous studies have shown that activation of intestinal T2Rs, receptors that are molecularly similar to the bitter taste receptor proteins of the oral cavity, result in the release of cholecystokinin [53]–[55], a well described regulator of meal size [56], [57]. It has been demonstrated that representative hydrolysable and condensed tannins activate specific bitter taste receptors [25], which have been shown to be present in the gut [58], perhaps the mechanism of PRP alteration of gut signaling is similar to the mechanism proposed to modify orosensory signaling. Specifically, the PRPs may bind to the tannic acid and subsequently disrupt its ability to interact with intestinal T2Rs.

The degree to which cystatin S may facilitate this process is an open question. There is some evidence that cystatins are related to bitter taste perception, however, there is no obvious mechanism for this relationship. Cystatin SN, a closely related protein to cystatin S, has been linked to the sensitivity to the bitter taste of caffeine in humans [23]. It has also been demonstrated that oral capsaicin [17] and quinine [18] stimulate the production of salivary cystatin S-like proteins suggesting that this class of proteins is generally responsive to aversive oral stimuli, but to our knowledge, this is the first suggestion that this protein is related to meal size.

## Conclusions

The current findings provide the first evidence that dietary-induced expression of salivary proteins correlates with feeding behavior. Because tannic acid containing diets are not consumed by animals incapable of upregulating PRPs [32], [33], we believe that the correlational relationship observed here is likely causal in nature. Our findings support and extend the work done by Glendinning [33] and Dsamou et al. [23] suggesting that salivary proteins can alter taste-guided ingestive responses. Taken together, our findings demonstrate that salivary proteins are involved in modulating both the orosensory and the postingestive feedback associated with chronic exposure to tannic acid diets. Understanding how the differential expression of these and other salivary proteins can modulate taste perception and the processing of postingestive signals represents a novel approach toward identifying individual differences in bitter taste acceptance.

## Supporting Information

Figure S1 **Depicts the HPLC chromatogram.** The solid line represents compounds detected in the tannic acid sample (2 µl, 5 mg/ml), the dotted line represents compounds detected in the PGG sample (0.25 µl, 5 mg/ml). Analysis of area under the curve confirms that 67% of the compounds in the tannic acid are the equivalent to PGG or larger in size, i.e. has a peak that is recorded later in minutes.
(TIF)

Figure S2 **Represents example gels of saliva samples fixed in Coomassie Brilliant Blue.** Equal volumes of saliva were resolved on 12% SDS-PAGE gels. Molecular mass markers (M) are on the left hand side of each gel. Under each gel is the Western blot of secretory immunoglobulin A (IgA) for each animal. Panel A represents samples collected from a single animal in the experimental group. Five control samples were collected during control diet (C) exposure on alternating days. Six samples were collected during tannic acid (T) exposure on alternating days. Panel B represents samples collected from a single animal in the control group. Seven control samples were collected during control (C) diet exposure on alternating days.
(TIF)

Table S1 **Liquid chromatography (LC) and mass spectrometry (MS) parameters used during protein sequencing.**
(DOCX)
