# Supplementary figures and images for: Induction of Salivary Proteins Modifies Measures of Both Orosensory and Postingestive Feedback during Exposure to a Tannic Acid Diet
Source: PLoS One. 2014 Aug 27;9(8):e105232. doi: 10.1371/journal.pone.0105232 (PMC4146545; doi:10.1371/journal.pone.0105232)

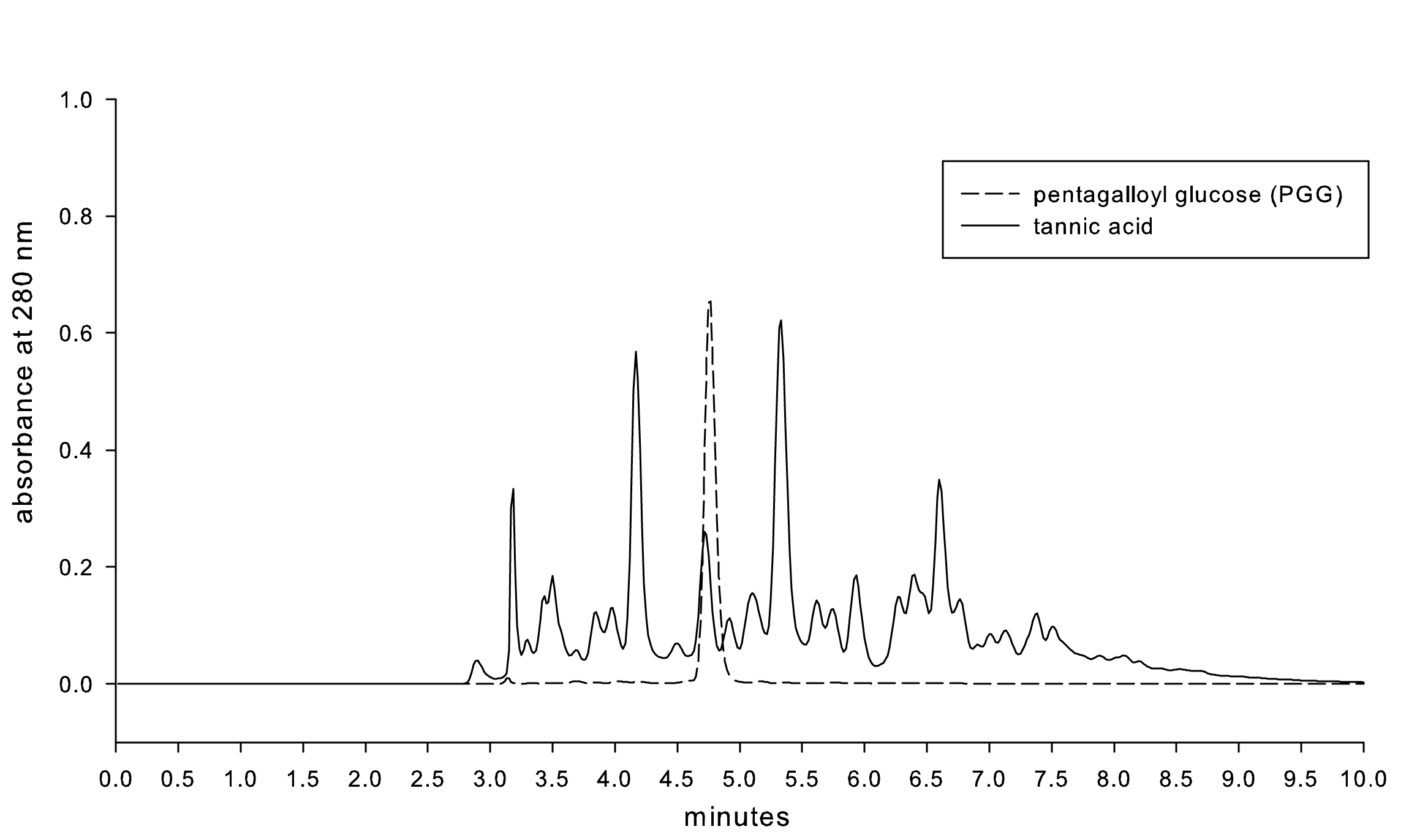

Supplement: Figure S1 — Depicts the HPLC chromatogram. The solid line represents compounds detected in the tannic acid sample (2 µl, 5 mg/ml), the dotted line represents compounds detected in the PGG sample (0.25 µl, 5 mg/ml). Analysis of area under the curve confirms that 67% of the compounds in the tannic acid are the equivalent to PGG or larger in size, i.e. has a peak that is recorded later in minutes. (TIF) [file pone.0105232.s001.tif]

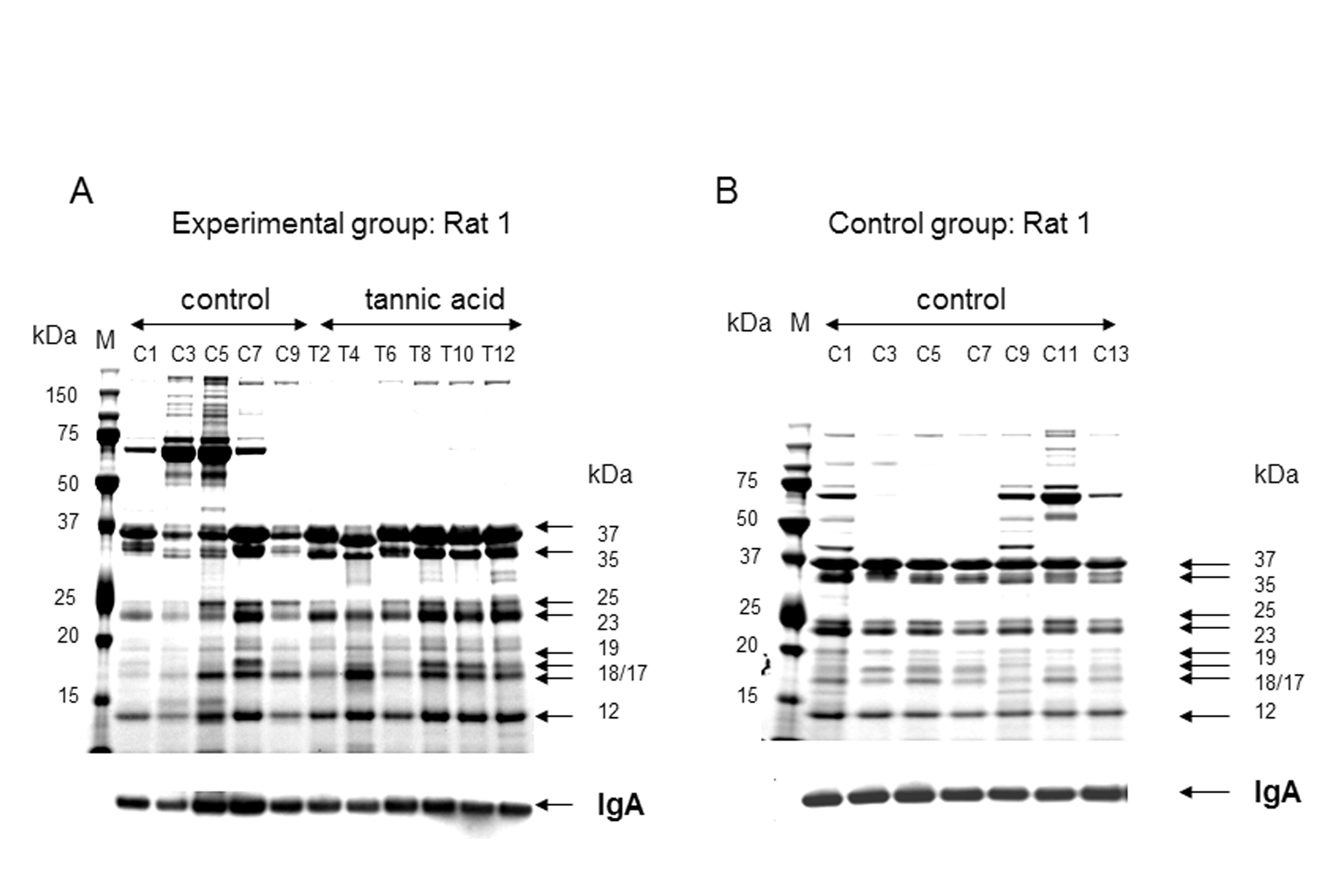

Supplement: Figure S2 — Represents example gels of saliva samples fixed in Coomassie Brilliant Blue. Equal volumes of saliva were resolved on 12% SDS-PAGE gels. Molecular mass markers (M) are on the left hand side of each gel. Under each gel is the Western blot of secretory immunoglobulin A (IgA) for each animal. Panel A represents samples collected from a single animal in the experimental group. Five control samples were collected during control diet (C) exposure on alternating days. Six samples were collected during tannic acid (T) exposure on alternating days. Panel B represents samples collected from a single animal in the control group. Seven control samples were collected during control (C) diet exposure on alternating days. (TIF) [file pone.0105232.s002.tif]
