# Supplementary material for: Induction of Salivary Proteins Modifies Measures of Both Orosensory and Postingestive Feedback during Exposure to a Tannic Acid Diet
Source: PLoS One. 2014 Aug 27;9(8):e105232. doi: 10.1371/journal.pone.0105232 (PMC4146545; doi:10.1371/journal.pone.0105232)
Supplement: Table S1 — Liquid chromatography (LC) and mass spectrometry (MS) parameters used during protein sequencing. (DOCX) [file pone.0105232.s003.docx]

Supplementary Table 1

Liquid chromatography (LC) and mass spectrometry (MS) parameters used during protein sequencing.

| LC Conditions | | MS Conditions | |
| --- | --- | --- | --- |
| LC System | Waters nanoAcquity UPLC | MS System | Waters Synapt G2 HD Mass Spectrometer |
| Column | Acquity UPLC BEH130 C_18_ | Ionization Mode | nESI Positive |
| Column Dimensions | 100 µm x 100 mm,  1.7 µm bead size | Capillary Voltage | 2.5 kV |
| Column Temp | 35°C | Cone Voltage | 35 V |
| Flow Rate | 0.880 µL/min | Nanoflow Gas | 1.0 Bar |
| Mobile Phase A | 0.1% Formic Acid (Aq) | Source Temp | 90°C |
| Mobile Phase B | 0.1% Formic Acid in Acetonitrile | Acquisition Range | 100-1990 m/z |
| Gradient | 3 - 40% B, 45 min | Collision Energies | MS 6 V, MS^E^ 25-60 V ramp |
